# AURKA rs2273535 T>A Polymorphism Associated With Cancer Risk: A Systematic Review With Meta-Analysis

**DOI:** 10.3389/fonc.2020.01040

**Published:** 2020-06-30

**Authors:** Shujie Wang, Jian Qi, Meiling Zhu, Meng Wang, Jinfu Nie

**Affiliations:** ^1^Anhui Province Key Laboratory of Medical Physics and Technology, Center of Medical Physics and Technology, Hefei Institutes of Physical Science, Chinese Academy of Sciences, Hefei, China; ^2^Hefei Cancer Hospital, Chinese Academy of Sciences, Hefei, China; ^3^Hefei Institute of Stem Cell and Regenerative Medicine, Guangzhou Institutes of Biomedicine and Health, Chinese Academy of Sciences, Hefei, China; ^4^Department of Oncology, Xinhua Hospital, School of Medicine, Shanghai Jiao Tong University, Shanghai, China

**Keywords:** meta-analysis, cell cycle, AURKA F31I, tumor, cancer risk

## Abstract

Aurora kinase A (AURKA) is a cell cycle regulatory serine/threonine kinase that promotes cell cycle progression. It plays an important role in regulating the transition from G2 to M phase during mitosis. The association between the AURKA rs2273535 T>A polymorphism and cancer risk has been investigated, but the results remain inconsistent. To get a more accurate conclusion, we conducted a comprehensive meta-analysis of 36 case-control studies, involving 22,884 cancer cases and 30,497 healthy controls. Crude odds ratios (ORs) and 95% confidence intervals (CIs) were calculated to determine the association of interest. Pooled analysis indicated that the AURKA rs2273535 T>A polymorphism increased the overall risk of cancer (homozygous: OR = 1.17, 95% CI = 1.04-1.33; recessive: OR = 1.15, 95% CI = 1.05-1.25; allele: OR = 1.07, 95% CI = 1.02-1.13). Stratification analysis by cancer type further showed that this polymorphism was associated with an increased breast cancer risk. This meta-analysis indicated that the AURKA rs2273535 T>A polymorphism was associated with an overall increased cancer risk, especially breast cancer. Further validation experiments are needed to strengthen our conclusion.

## Introduction

Aurora kinase A (AURKA) is a cell-cycle regulatory serine/threonine kinase that promotes cell cycle progression (1). AURKA is expressed in proliferating cells, especially in the G2 and mitotic phases of the cell cycle. It has various roles in promoting cell division, including the establishment of the mitotic spindle and centrosome separation (1). The AURKA gene is located at chromosomal locus 20q13.2 according to the HUGO Gene Nomenclature Committee (HGNC), and is often amplified in cancers (2–4). AURKA amplification has been found in certain tumor types, including colorectal, leukemia, and pancreatic cancers (3, 5, 6). Overexpression of AURKA can promote cellular transformation and may potentiate the activity of other oncogenes, such as RAS, further promoting tumorigenesis (7).

AURKA rs2273535 T>A, also known as F31I or Phe31Ile, is caused by a T-to-A transversion at position 91 in the AURKA coding sequence. This single nucleotide polymorphism is located in the Aurora Box1 (aa 5-40) motif, which belongs to the NH2-terminal region of aurora-A. This motif is thought to act on ubiquitin-based proteolysis (8).

Up until now, there have been many studies that have investigated the association between the AURKA F31I polymorphism and the risk of different types of cancer in different populations. However, the results are inconsistent, most likely because the sample size is relatively small in each published study, and the impact of the polymorphism on cancer risk might be small. Therefore, we conducted a comprehensive meta-analysis by identifying as many relevant articles as possible to identify evidence for the link between the AURKA F31I polymorphism and cancer risk.

## Materials and Methods

### Search Strategy

Two authors (JQ and SW) conducted the search for relevant articles before December 2018, using the following terms, “AURKA or Aurora Kinase A,” “tumor or cancer or carcinoma or neoplasm,” and “polymorphism or single-nucleotide polymorphism (SNP) or variant,” in the PubMed, EMBASE, CNKI, WANFANG, and Vip databases. We also examined the references of the retrieved publications for additional eligible studies.

### Eligibility Criteria

Publications retrieved from the various databases were assessed for eligibility according to the following criteria: (1) the publication was published in English or Chinese; (2) the publication evaluated the association between the AURKA gene rs2273535 polymorphism and cancer risk; (3) the publication was a case-control study; the publication with the largest number of individuals was selected if studies had duplicate subjects. In addition, publications were excluded according to the following criteria: (1) genotype data included were not sufficient to calculate an odds ratio (OR) and 95% confidence interval (CI); (2) only survival data was included; (3) the genotype frequency distribution departed from Hardy-Weinberg equilibrium (HWE) in the controls.

### Data Extraction

The following information was independently extracted by two authors (SW and JQ): first author name, year of publication, cancer type, region, ethnicity, genotyping method, source of controls (hospital-based, population-based, and mixed), the genotype counts of cases, and controls for the investigated polymorphism.

### Quality Assessment

Two authors (SW and JQ) independently assessed the quality of the included studies according to the Strengthening the Reporting of Observational studies in Epidemiology (STROBE) quality scoring system (9). Forty evaluation items related to quality assessment were used in meta-analysis, with scores ranging from 0 to 40. Based on the STROBE score, the included studies were classified as low quality (0-19), moderate quality (20-29), and high quality (30-40) (10). If the studies were of low quality, moderate, or high quality, they were considered to be at high, moderate or low risk of bias, respectively. If two authors had contradictory information, a third author (MW) was consulted.

### Statistical Analysis

Pooled ORs and 95% CIs were evaluated to assess the relationship between the AURKA rs2273535 T>A polymorphism and overall cancer risk under the heterozygous (AT vs. TT), homozygous (AA vs. TT), dominant (AT+AA vs. TT), recessive (AA vs. AT+TT), and allele contrast (A vs. T) models. We conducted stratification analyses by ethnicity, cancer type (“others”: one cancer type was investigated in less than 3 studies), study design (“mixed”: the source of controls contained both hospital-based and population-based subjects) and risk of bias. The Chi square-based Q-test was used to calculate the heterogeneity among studies. A random-effect model was adopted when *P* < 0.1 (heterogeneity). Otherwise, a fixed-effect model was adopted (11). Funnel plots were used to evaluate potential publication bias (12). All data analyses were performed using R software. All of the P values were two-tailed; *P* < 0.05 indicated statistical significance.

## Results

### Literature Search

**Figure 1:**
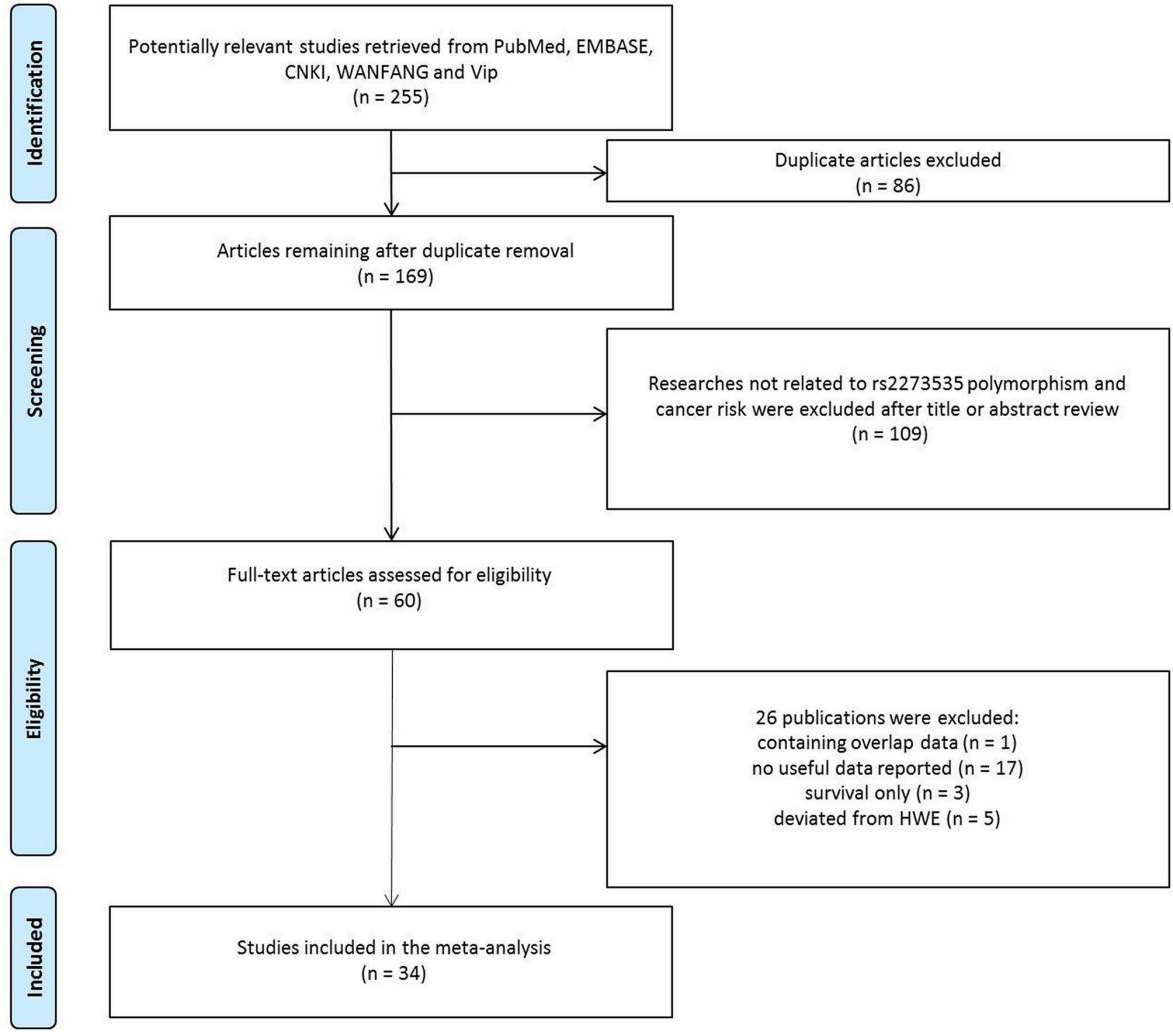
Flowchart of included publications.

As shown in Figure 1, 255 potentially relevant studies were selected from the PubMed, CNKI, EMBASE, WANFANG, and Vip databases. We excluded 86 duplicate articles and 109 publications not investigating the association between the AURKA gene rs2273535 polymorphism and cancer risk after reviewing titles and abstracts. Then, the full texts of the remaining articles were evaluated. One publication (13) was removed for containing overlapping data. We also excluded 17 publications (14–30) because no useful data was reported to calculate ORs and 95% CIs. In addition, we eliminated 3 publications (31–33) presenting survival data only. Lastly, we excluded 5 publications (34–38) due to deviation from the HWE. Overall, 34 publications with a total of 22,884 cancer cases and 30,497 healthy controls were included in the meta-analysis.

### Description and Quality of the Studies

The 34 publications actually consisted of 36 case-control studies, because 2 publications included 2 individual studies. The characteristics of these studies were shown in Supplemental Table 1. Among these publications, 12 focused on breast cancer (39–50), 5 on gastric cancer (51–55), 3 on colorectal cancer (56–58), 2 on esophageal (59, 60), 2 on liver cancer (61, 62), and 2 on oral cancer (63, 64). Moreover, there was only 1 study for each of the following cancers: lung cancer (65), neuroblastoma cancer (66), ovarian cancer (67), prostate cancer (68), renal cancer (69), urinary tract urothelial cancer (70), uterine cancer (71), bladder cancer (70), and endometrial cancer (72). As evident in Table 1, among these case-control studies, 2 of them had low risk of bias, 31 had moderate risk of bias, while 3 had high risk of bias. The shortcomings of low risk of bias research mainly focused on the lack of some descriptions in the results section (describing the numbers of individuals at each stage of the study, reasons for non-participation at each stage and a flow diagram) and methods section (describing comparability of assessment methods, address potential sources of bias, the estimation of the study size). In addition to the shortcomings involved in the low-risk bias study, the moderate-risk bias study also lacked the description in the methods section(explaining how missing data were addressed, how missing data were addressed and how quantitative variables were handled in the analyses), results section(indicating the number of participants with missing data and if relevant, considering translating estimates of relative risk into absolute risk for a meaningful time period) and discussion section (reporting category boundaries when continuous variables were categorized). High-risk bias study not only included the above-mentioned defects, but included the lack of giving the sources and methods of case ascertainment and control selection, the rationale for the choice of cases and controls and matching criteria and the number of controls, clearly defining all outcomes, exposures, predictors, potential confounders, effect modifiers and describing any sensitivity analyses in the methods section. Finally, this meta-analysis contained 19 hospital-based and 15 population-based studies.

**Table 1:** The risk of bias assessment.

**Name**	**Year**	**STROBE score**	**Risk of bias**
Miao	2004	22	Moderate
Zhiyu Bao	2017	28	Moderate
Zheng	2013	27	Moderate
Zhang	2006	21	Moderate
Ying-ChuLin	2017	23	Moderate
Ying-ChuLin	2017	23	Moderate
Xiaoyan Zhou	2018	24	Moderate
Webb	2006	20	Moderate
Vidarsdottir	2007	23	Moderate
Tchatchou	2007	21	Moderate
Sun	2004	20	Moderate
Shi	2011	27	Moderate
Shan Li	2015	25	Moderate
Ruan	2011	26	Moderate
Nicholas J. Taylor	2015	30	High
Nicholas J. Taylor	2015	30	High
Ming Zhao	2014	14	Low
Milam	2007	22	Moderate
Marie-Genica	2010	26	Moderate
Lo	2005	23	Moderate
Li-Yuan Zheng	2015	28	Moderate
Li Chen	2005	12	Low
Jue Tang	2018	23	Moderate
Ju	2006	21	Moderate
Hammerschmied	2007	20	Moderate
Guenard	2009	21	Moderate
Gu	2007	27	Moderate
Feik	2009	22	Moderate
Cox	2006	23	Moderate
Chi-Pin Lee	2015	25	Moderate
Chia-Hsuan Chou	2017	16	Low
Chen	2007	20	Moderate
Chen	2009	21	Moderate
Bin Wang	2018	25	Moderate
Aner Mesic	2016	22	Moderate
Andrés López-Cortés	2017	23	Moderate

### Meta-Analysis Results

**Figure 2:**
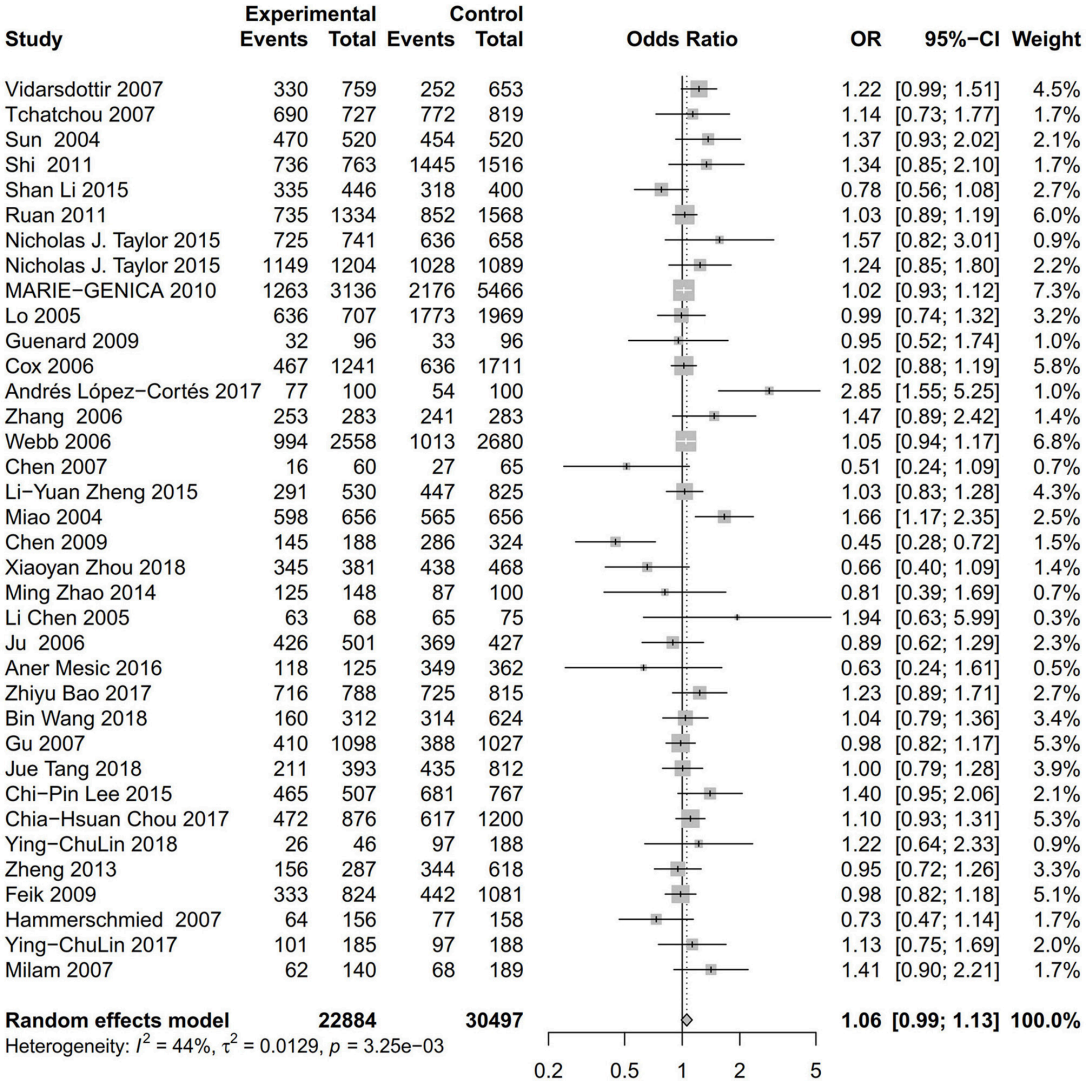
Forest plot of the association between the AURKA rs2273535 T>A polymorphism and overall cancer risk under the dominant model (AT+AA vs. TT).

**Figure 3:**
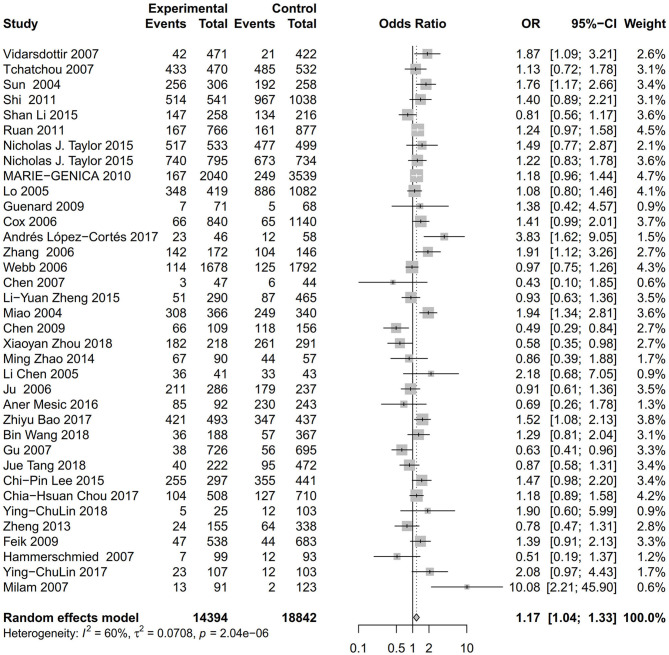
Forest plot of the association between the AURKA rs2273535 T>A polymorphism and overall cancer risk under the homozygous model (AA vs. TT).

**Figure 4:**
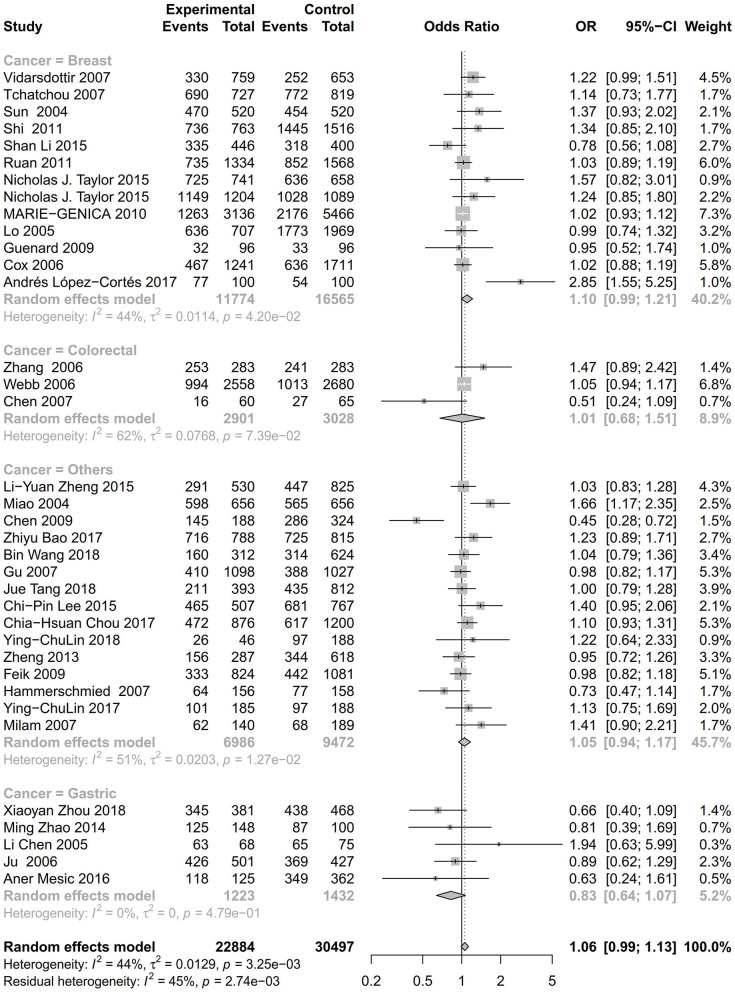
Stratification analysis of the association between the AURKA rs2273535 T>A polymorphism and cancer risk by cancer type under the dominant model (AT+AA vs. TT).

**Figure 5:**
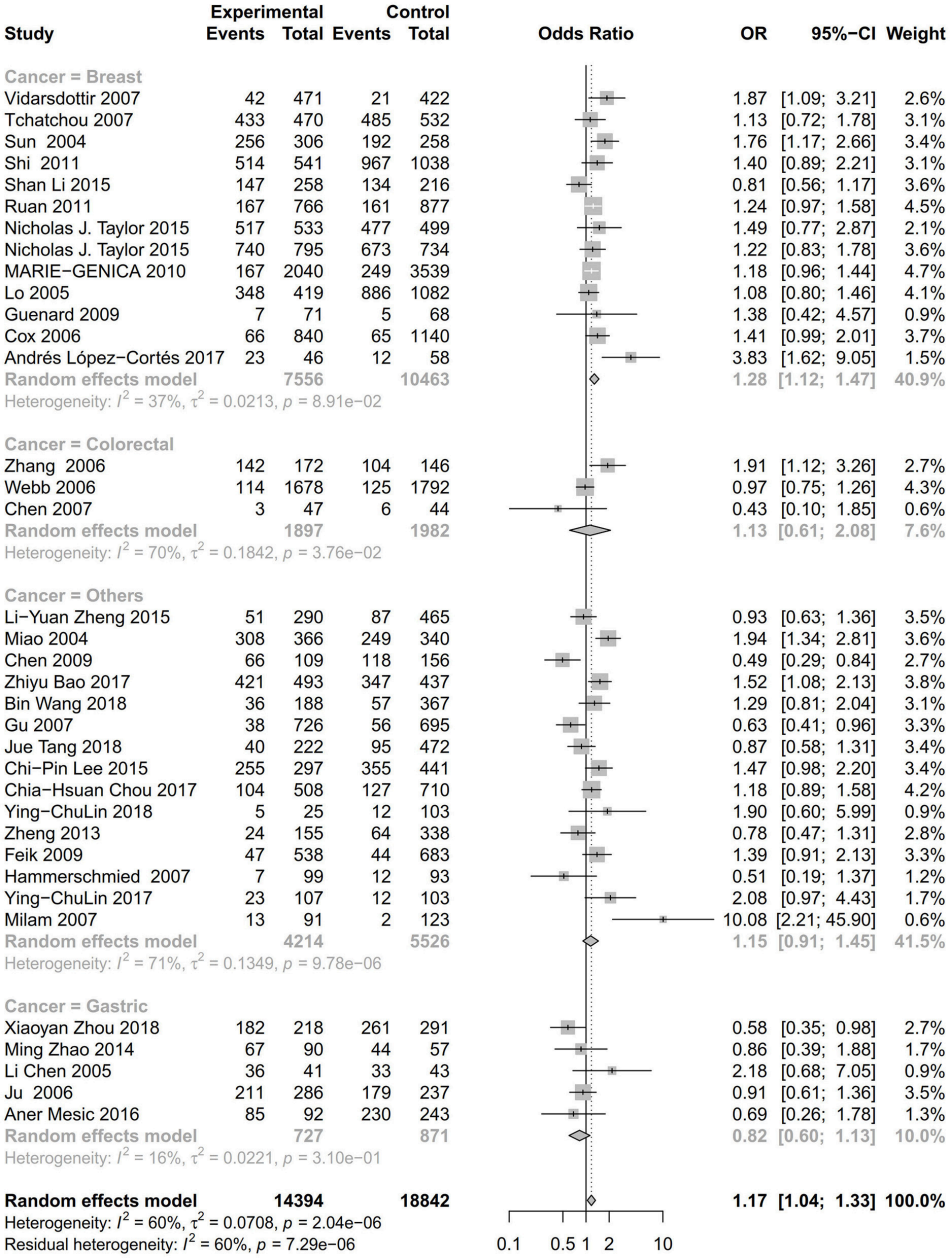
Stratification analysis of the association between the AURKA rs2273535 T>A polymorphism and cancer risk by cancer type under the homozygous model (AA vs. TT).

**Figure 6:**
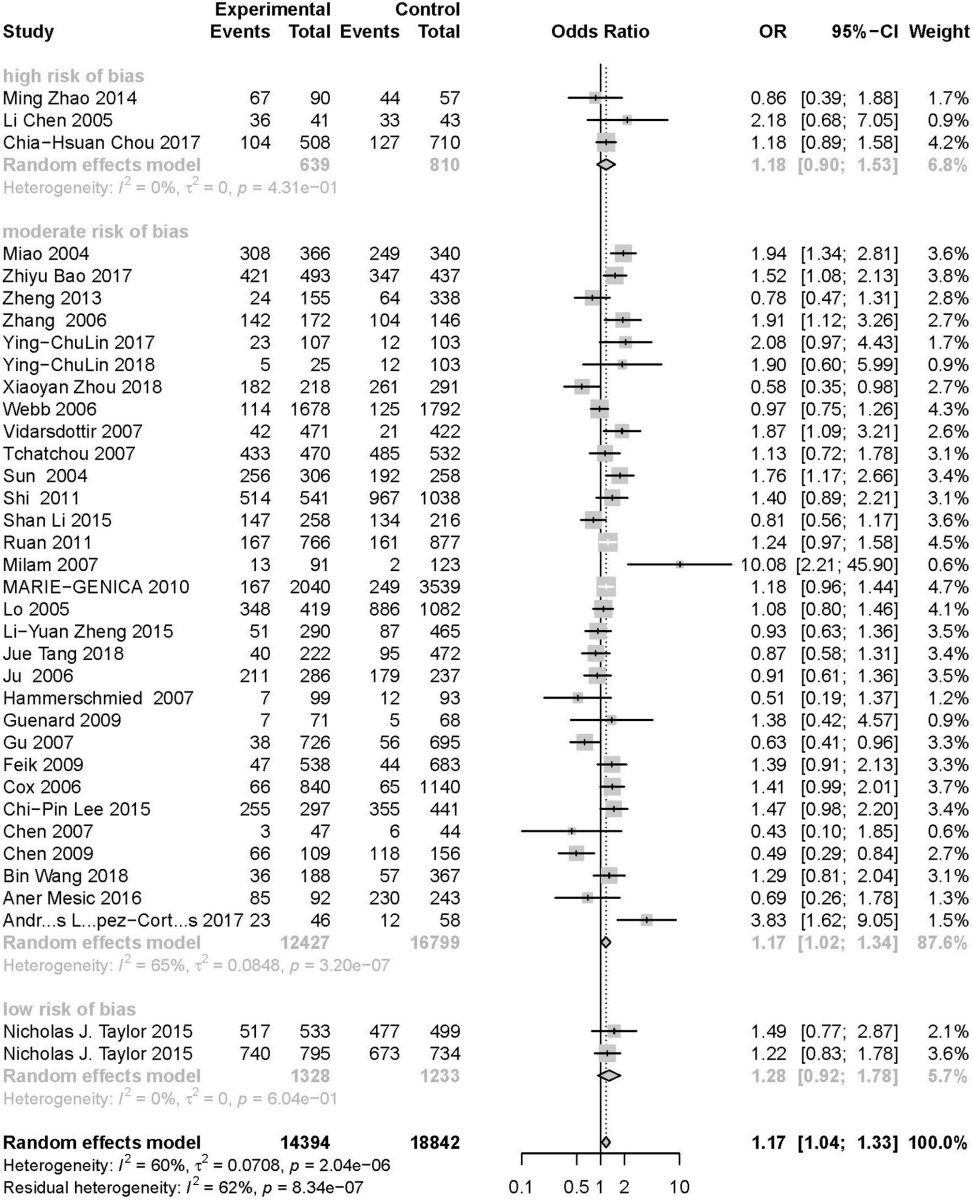
Stratification analysis of the association between the AURKA rs2273535 T>A polymorphism and cancer risk by risk of bias under the homozygous model (AA vs. TT).

As evident in Table 2, Figures 2, 3, there is significant inter-study heterogeneity under all the genetic models; thus, we used a random-effect model. After calculating crude ORs and 95% CIs, we found that the AURKA gene rs2273535 T>A polymorphism was associated with increased overall cancer susceptibility (homozygous: OR = 1.17, 95% CI = 1.04-1.33; recessive: OR = 1.15, 95% CI = 1.05-1.25; allele: OR = 1.07, 95% CI = 1.02-1.13). Figures 2, 3 depicted forest plot of the association between the AURKA rs2273535 T>A polymorphism and overall cancer risk under the dominant and homozygous model. We performed stratification analyses by cancer type, ethnicity, study design and risk of bias. Stratification analysis further indicated that the AURKA gene rs2273535 T>A polymorphism was associated with increased risk of breast cancer (homozygous: OR = 1.28, 95% CI = 1.12-1.47; recessive: OR = 1.17, 95% CI = 1.05-1.31; allele: OR = 1.09, 95% CI = 1.02-1.17). Figures 4, 5 show stratification analysis of the association between the AURKA rs2273535 T>A polymorphism and cancer risk by cancer type under the dominant and homozygous model. We also checked the association in the Asian population. Interestingly, we only observed significant associations in Asians (recessive: OR = 1.17, 95% CI = 1.05-1.32; allele: OR = 1.09, 95% CI = 1.01-1.18). Moreover, the association remained significant in the subgroups with population-based studies (homozygous: OR = 1.25, 95% CI = 1.07-1.46; recessive: OR = 1.19, 95% CI = 1.07-1.33; allele: OR = 1.10, 95% CI = 1.03-1.19) and moderate risk of bias (homozygous: OR = 1.17, 95% CI = 1.02-1.34; recessive: OR = 1.17, 95% CI = 1.06-1.29; allele: OR = 1.08, 95% CI = 1.02-1.14). Figure 6 revealed stratification analysis of the association between the AURKA rs2273535 T>A polymorphism and cancer risk by risk of bias under the homozygous model.

**Table 2:** The association between the AURKA rs2273535 T>A polymorphism and cancer risk in the meta-analysis.

**Variables**	**No. of studies**	**Homozygous**		**Heterozygous**		**Recessive**		**Dominant**		**Allele**	
		**AA vs. TT**		**AT vs. TT**		**AA vs. AT+TT**		**AT+AA vs. TT**		**A vs. T**	
		**OR (95% CI)**	***P*^**het**^**	**OR (95% CI)**	***P*^**het**^**	**OR (95% CI)**	***P*^**het**^**	**OR (95% CI)**	***P*^**het**^**	**OR (95% CI)**	***P*^**het**^**
All	36	1.17 (1.04-1.33)	0.000	1.02 (0.97-1.06)	0.096	1.15 (1.05-1.25)	0.000	1.06 (0.99-1.13)	0.003	1.07 (1.02-1.13)	0.000
**Cancer type**											
Breast	13	1.28 (1.12-1.47)	0.089	1.02 (0.96-1.08)	0.098	1.17 (1.05-1.31)	0.008	1.10 (0.99-1.21)	0.042	1.09 (1.02-1.17)	0.006
Colorectal	3	1.13 (0.61-2.08)	0.038	1.05 (0.93-1.17)	0.258	1.15 (0.67-1.98)	0.012	1.01 (0.68-1.51)	0.074	1.05 (0.73-1.50)	0.003
Gastric	5	0.82 (0.60-1.13)	0.310	0.83 (0.63-1.09)	0.654	0.99 (0.79-1.24)	0.140	0.83 (0.64-1.07)	0.479	0.96 (0.80-1.14)	0.132
Others	15	1.15 (0.91-1.45)	0.000	1.02 (0.95-1.10)	0.130	1.14 (0.96-1.36)	0.000	1.05 (0.94-1.17)	0.013	1.08 (0.98-1.18)	0.000
**Ethnicity**											
Caucasian	14	1.15 (0.95-1.39)	0.007	1.02 (0.96-1.08)	0.595	1.11 (0.96-1.28)	0.007	1.04 (0.98-1.10)	0.357	1.05 (0.99-1.11)	0.090
Asian	20	1.15 (0.98-1.34)	0.000	1.00 (0.93-1.07)	0.194	1.17 (1.05-1.32)	0.000	1.04 (0.94-1.15)	0.011	1.09 (1.01-1.18)	0.000
**Strobe score**											
30-40	2	1.28 (0.92-1.78)	0.604	1.39 (0.99-1.95)	0.391	0.95 (0.83-1.08)	0.419	1.31 (0.95-1.82)	0.541	1.00 (0.89-1.12)	0.536
20-29	31	1.17 (1.02-1.34)	0.000	1.01 (0.95-1.07)	0.097	1.17 (1.06-1.29)	0.000	1.05 (0.97-1.12)	0.002	1.08 (1.02-1.14)	0.000
0-19	3	1.18 (0.90-1.53)	0.431	1.07 (0.90-1.28)	0.524	1.15 (0.92-1.45)	0.762	1.10 (0.93-1.30)	0.443	1.09 (0.97-1.23)	0.497
**Design**											
HB	19	1.10 (0.91-1.33)	0.000	1.01 (0.95-1.08)	0.664	1.10 (0.95-1.27)	0.000	1.02 (0.94-1.11)	0.157	1.05 (0.97-1.13)	0.000
PB	16	1.25 (1.07-1.46)	0.002	1.02 (0.96-1.08)	0.009	1.19 (1.07-1.33)	0.001	1.10 (0.99-1.22)	0.001	1.10 (1.03-1.19)	0.000

*OR, odd ratio; CI, confidence interval; Het, heterogeneity*.

### Publication Bias

**Figure 7:**
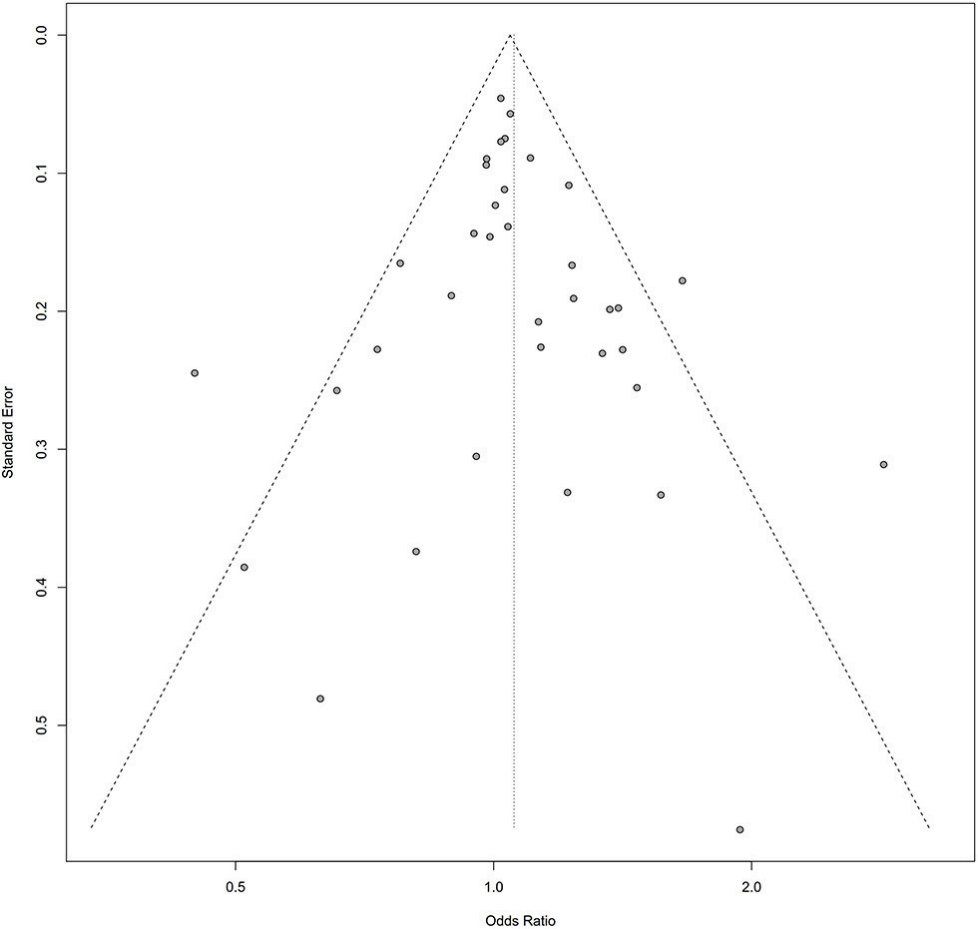
Funnel plot of the association between the AURKA rs2273535 T>A polymorphism and overall cancer risk under the dominant model (AT+AA vs. TT).

**Figure 8:**
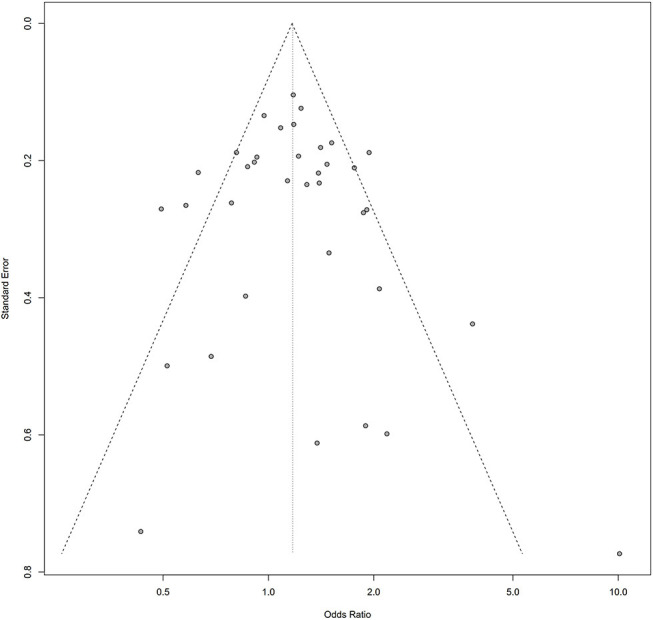
Funnel plot of the association between the AURKA rs2273535 T>A polymorphism and overall cancer risk under the homozygous model (AA vs. TT).

Symmetry in the funnel plots (Figures 7, 8) suggested that there was no significant publication bias in this meta-analysis (homozygous: P = 0.585; heterozygous: P = 0.939; recessive: P = 0.586; dominant: P = 0.546; allele: P = 0.657).

## Discussion

AURKA is a key factor in regulating thetransition from G2 to M phase during mitosis. The AURKA protein includes a 129-amino acid N-terminal domain that facilitates AURKA nuclear-translocation during mitosis and a 274-amino acid C-terminal kinase catalytic domain (73). AURKA has been reportedly associated with poor prognosis in medulloblastoma and over-expression in various types of cancer (74). AURKA protein amplification and over-expression in breast and other tumors is related to centrosomal amplification, dysfunction of cytokinesis, and aneuploidy. Based on genetic mapping studies, AURKA is a potential genetic target for cancer therapy (16). The AURKA F31I polymorphism (T>A)(phenylalanine (Phe)> isoleucine (Ile)) is related to cellular transformation and distinctly enhances chromosomal instability (75). This polymorphism can also cause an obstruction in p53 binding and decreased degradation of AURKA by changing the activity of the AURKA box 1 (16). Research has shown that the stabilized over-expression of AURKA results in centrosomal amplification, abnormal cytokinesis, chromosomal instability, and the promotion of tumorigenesis. Numerous studies have been performed to explore the association between the rs2273535 polymorphism and the risk of various types of cancer. López-Cortés et al. (50) carried out a study in 2018 to investigate the role of single nucleotide polymorphism AURKA T91A (rs2273535) in a high altitude Ecuadorian Mestizo population consisting of 100 patients and 100 controls, and found a significant relationship between the rs2273535 genotype and a higher risk of breast cancer development. This association was confirmed in different types of cancer, including hepatocellular carcinoma (HCC) by Bao et al. (62) with 788 cases and 815 controls, urinary tract urothelial cancer by Lin et al. (70) with 185 cases and 188 controls, gastric cancer by Zhou et al. (51) with 381 cases and 468 controls, as well as other types of cancer. However, opposing results were also frequently reported. A case-control study containing 501 prostate cancer and 427 control subjects conducted by Feik et al. (68) revealed that the AURKA rs2273535 polymorphism was not found to be related to prostate cancer risk. Additionally, Ju et al. (54) reported that this polymorphism was not related to gastric cancer susceptibility, by studying 501 cases and 427 controls. Tang et al. (66) selected 393 cases and 812 controls, and the results indicated that none of the AURKA polymorphisms were associated with neuroblastoma susceptibility in two distinct Chinese populations. Several meta-analyses have also been conducted, and unfortunately the results were still inconclusive (76–80). In this meta-analysis, the association between the AURKA gene rs2273535 T>A polymorphism and cancer risk based on 36 eligible case-control studies, with a total of 22,884 cancer cases and 30,497 healthy controls, was estimated. Among these case-control studies, 2 of them had low risk of bias, 31 had moderate risk of bias, while 3 had high risk of bias. Most quality scores of the included studies were higher than 20 (low to moderate risk of bias). Overall, our results indicated that this polymorphism might increase the overall risk of cancer, especially breast cancer.

However, there were some limitations in this meta-analysis. First, only publications written in Chinese or English were selected. Second, the number of studies for certain cancer types was inadequate, such as colorectal cancer (<5 studies). There were 3 included studies having high risk of bias (0 ≤ STROBE score ≤ 19); further studies with low risk of bias are needed to validate the true association. In addition, other factors may also influence cancer risk, such as age and living habits. Our findings might suffer from potential confounding bias due to the lack of original data. Taken together, the results should be interpreted with caution.

To conclude, this meta-analysis suggests that the AURKA gene rs2273535 T>A polymorphism is significantly associated with an overall increased cancer risk, especially breast cancer. In the research, most of the included studies had low to moderate risk of bias. Future well-designed, large-scale studies that report upon the association of the rs2273535 polymorphism and cancer, in multiple cancer types, are required to validate the findings of this study.

## Data Availability Statement

The datasets generated for this study can be found in the article/Supplementary Material.

## Author Contributions

SW, MW, MZ and JN conceived and designed the study. SW, JQ, and MW conducted the literature searches, extracted the data, analyzed the data and prepared the figures and tables. SW, MW, and JQ wrote the draft of manuscript. SW, MW, and JN revised the manuscript. All authors contributed to the article and approved the submitted version.

## Conflict of Interest

The authors declare that the research was conducted in the absence of any commercial or financial relationships that could be construed as a potential conflict of interest.
