# Supplementary material for: AURKA rs2273535 T>A Polymorphism Associated With Cancer Risk: A Systematic Review With Meta-Analysis
Source: Front Oncol. 2020 Jun 30;10:1040. doi: 10.3389/fonc.2020.01040 (PMC7357424; doi:10.3389/fonc.2020.01040)
Supplement: Supplementary file 1 [file Table_1.DOCX]

Supplemental Table 1. Characteristics of included studies in the meta-analysis.

| **Name** | **Year** | **Cancer type** | **Region** | **Ethnicity** | **Design** | **Genotype method** | **Case** | | | | **Control** | | | | **MAF** | **HWE** |
| --- | --- | --- | --- | --- | --- | --- | --- | --- | --- | --- | --- | --- | --- | --- | --- | --- |
|  |  |  |  |  |  |  | **TT** | **AT** | **AA** | **All** | **TT** | **AT** | **AA** | **All** |  |  |
| Miao | 2004 | Esophageal | China | Asian | PB | PCR-RFLP | 58 | 290 | 308 | 656 | 91 | 316 | 249 | 656 | 0.62 | 0.578 |
| Zhiyu Bao | 2017 | Liver | China | Asian | PB | PCR-RFLP | 72 | 295 | 421 | 788 | 90 | 378 | 347 | 815 | 0.66 | 0.394 |
| Zheng | 2013 | Ovarian | China | Asian | PB | Taqman | 131 | 132 | 24 | 287 | 274 | 280 | 64 | 618 | 0.33 | 0.595 |
| Zhang | 2006 | Colorectal | China | Asian | PB | PCR-RFLP | 30 | 111 | 142 | 283 | 42 | 137 | 104 | 283 | 0.61 | 0.712 |
| Ying-ChuLin | 2017 | Urinary tract urothelial | China | Asian | HB | TaqMan | 84 | 78 | 23 | 185 | 91 | 85 | 12 | 188 | 0.29 | 0.150 |
| Ying-ChuLin | 2017 | bladder | China | Asian | HB | TaqMan | 20 | 21 | 5 | 46 | 91 | 85 | 12 | 188 | 0.29 | 0.150 |
| Xiaoyan Zhou | 2018 | Gastric | China | Asian | HB | scanTM 48-Plex SNP Kit | 36 | 163 | 182 | 381 | 30 | 177 | 261 | 468 | 0.75 | 1.000 |
| Webb | 2006 | Colorectal | UK | Caucasian | HB | ISBA | 1564 | 880 | 114 | 2558 | 1667 | 888 | 125 | 2680 | 0.21 | 0.641 |
| Vidarsdottir | 2007 | Breast | Iceland | Caucasian | HB | PCR–RFLP | 429 | 288 | 42 | 759 | 401 | 231 | 21 | 653 | 0.21 | 0.0754 |
| Tchatchou | 2007 | Breast | German | Caucasian | HB | Taqman | 37 | 257 | 433 | 727 | 47 | 287 | 485 | 819 | 0.77 | 0.621 |
| Sun | 2004 | Breast | China | Asian | HB | PCR-RFLP | 50 | 214 | 256 | 520 | 66 | 262 | 192 | 520 | 0.62 | 0.115 |
| Shi | 2011 | Breast | Sweden | Caucasian | PB | Taqman | 27 | 222 | 514 | 763 | 71 | 478 | 967 | 1516 | 0.80 | 0.236 |
| Shan Li | 2015 | Breast | China | Asian | HB | PCR-RFLP | 111 | 188 | 147 | 446 | 82 | 184 | 134 | 400 | 0.57 | 0.221 |
| Ruan | 2011 | Breast | China | Asian | PB | Taqman | 599 | 568 | 167 | 1334 | 716 | 691 | 161 | 1568 | 0.32 | 0.768 |
| Nicholas J. Taylor | 2015 | Breast | American | African American | PB | Assay | 16 | 208 | 517 | 741 | 22 | 159 | 477 | 658 | 0.85 | 0.0704 |
| Nicholas J. Taylor | 2015 | Breast | American | Caucasian | PB | Assay | 55 | 409 | 740 | 1204 | 61 | 355 | 673 | 1089 | 0.78 | 0.134 |
| Ming Zhao | 2014 | Gastric | China | Asian | HB | PCR-RFLP | 23 | 58 | 67 | 148 | 13 | 43 | 44 | 100 | 0.66 | 0.655 |
| Milam | 2007 | Uterine | USA | Caucasian | HB | Taqman | 78 | 49 | 13 | 140 | 121 | 66 | 2 | 189 | 0.19 | 0.0510 |
| MARIE-GENICA | 2010 | Breast | German | Caucasian | PB | PCR-RFLP | 1873 | 1096 | 167 | 3136 | 3290 | 1927 | 249 | 5466 | 0.22 | 0.118 |
| Lo | 2005 | Breast | China | Asian | HB | Taqman | 71 | 288 | 348 | 707 | 196 | 887 | 886 | 1969 | 0.68 | 0.230 |
| Li-Yuan Zheng | 2015 | endometrial | China | Asian | PB | TaqMan | 239 | 240 | 51 | 530 | 378 | 360 | 87 | 825 | 0.32 | 0.938 |
| Li Chen | 2005 | Gastric | China | Asian | HB | PCR-RFLP | 5 | 27 | 36 | 68 | 10 | 32 | 33 | 75 | 0.65 | 0.796 |
| Jue Tang | 2018 | Neuroblastoma | China | Asian | HB | TaqMan | 182 | 171 | 40 | 393 | 377 | 340 | 95 | 812 | 0.33 | 0.172 |
| Ju | 2006 | Gastric | Korea | Asian | HB | MassARRAY | 75 | 215 | 211 | 501 | 58 | 190 | 179 | 427 | 0.64 | 0.522 |
| Hammerschmied | 2007 | Renal | German | Caucasian | HB | PCR–RFLP | 92 | 57 | 7 | 156 | 81 | 65 | 12 | 158 | 0.28 | 0.847 |
| Guenard | 2009 | Breast | Canada | Caucasian | NA | Sequencing | 64 | 25 | 7 | 96 | 63 | 28 | 5 | 96 | 0.20 | 0.520 |
| Gu | 2007 | Lung | USA | Caucasian | HB | Taqman | 688 | 372 | 38 | 1098 | 639 | 332 | 56 | 1027 | 0.22 | 0.142 |
| Feik | 2009 | Prostate | Australia | Caucasian | HB | Taqman | 491 | 286 | 47 | 824 | 639 | 398 | 44 | 1081 | 0.22 | 0.0646 |
| Cox | 2006 | Breast | USA | Caucasian | PB | TaqMan | 774 | 401 | 66 | 1241 | 1075 | 571 | 65 | 1711 | 0.20 | 0.297 |
| Chi-Pin Lee | 2015 | Oral | China | Asian | HB | TaqMan | 42 | 210 | 255 | 507 | 86 | 326 | 355 | 767 | 0.68 | 0.408 |
| Chia-Hsuan Chou | 2017 | Oral | China | Asian | PB | TaqMan | 404 | 368 | 104 | 876 | 583 | 490 | 127 | 1200 | 0.31 | 0.118 |
| Chen | 2007 | Colorectal | USA | Caucasian | HB | Sequencing | 44 | 13 | 3 | 60 | 38 | 21 | 6 | 65 | 0.25 | 0.317 |
| Chen | 2009 | Esophageal | China | Asian | PB | PCR-RFLP | 43 | 79 | 66 | 188 | 38 | 168 | 118 | 324 | 0.62 | 0.0796 |
| Bin Wang | 2018 | Liver | China | Asian | PB | TaqMan | 152 | 124 | 36 | 312 | 310 | 257 | 57 | 624 | 0.30 | 0.770 |
| Aner Mesic | 2016 | Gastric | Slovenian | Caucasian | PB | TaqMan | 7 | 33 | 85 | 125 | 13 | 119 | 230 | 362 | 0.80 | 0.629 |
| Andrés López-Cortés | 2017 | Breast | Ecuadorian | Mixed | PB | sequence | 23 | 54 | 23 | 100 | 46 | 42 | 12 | 100 | 0.33 | 0.657 |

Abbreviations: MAF, minor allele frequency; HWE, Hardy-Weinberg equilibrium; HB, hospital-based; PB, population-based; PCR-RFLP, polymerase chain reaction-restriction fragment length polymorphism.
